# Measurement of Gas-Liquid Two-Phase Flow in Micro-Pipes by a Capacitance Sensor

**DOI:** 10.3390/s141222431

**Published:** 2014-11-26

**Authors:** Haifeng Ji, Huajun Li, Zhiyao Huang, Baoliang Wang, Haiqing Li

**Affiliations:** State Key Laboratory of Industrial Control Technology, Department of Control Science and Engineering, Zhejiang University, Hangzhou 310027, China; E-Mails: lihuajun@zju.edu.cn (H.L.); blwang@iipc.zju.edu.cn (B.W.); hqli@iipc.zju.edu.cn (H.L.)

**Keywords:** capacitance sensors, two-phase flow, micro-pipe, slug flow, voidage, velocity, cross-correlation

## Abstract

A capacitance measurement system is developed for the measurement of gas-liquid two-phase flow in glass micro-pipes with inner diameters of 3.96, 2.65 and 1.56 mm, respectively. As a typical flow regime in a micro-pipe two-phase flow system, slug flow is chosen for this investigation. A capacitance sensor is designed and a high-resolution and high-speed capacitance measurement circuit is used to measure the small capacitance signals based on the differential sampling method. The performance and feasibility of the capacitance method are investigated and discussed. The capacitance signal is analyzed, which can reflect the voidage variation of two-phase flow. The gas slug velocity is determined through a cross-correlation technique using two identical capacitance sensors. The simulation and experimental results show that the presented capacitance measurement system is successful. Research work also verifies that the capacitance sensor is an effective method for the measurement of gas liquid two-phase flow parameters in micro-pipes.

## 1. Introduction

Two-phase flow systems widely exist in industrial process. The parameter measurement of such systems is always one of the most important and difficult research topics [1,2]. Now more and more two-phase flow systems in micro-channel or mini-channel forms are appearing in chemical reactors, exchangers, cooling processes and many other systems. The advantages of increased heat and mass transfer in small dimensions and extremely large surface-to-volume ratio are demonstrated with model gas, liquid and multiphase reaction systems [3–6]. The study of the characteristics in such applications requires the measurement of two-phase flow parameters, including flow patterns, pressure, temperature, phase velocity and voidage, *etc.* However, the flow characteristics in small-scale pipes make the parameter measurement more difficult than in normal scale pipes, so what parameter measurement techniques can be applied and how to apply them in micro-pipe two-phase flow research becomes a critical problem.

Different parameter measurement techniques have been applied to measure micro-channel two-phase flow, including visualization, optical and electrical measurement methods. Triplett *et al.* studied gas-water two-phase flow in micro-pipes with 1.1 mm and 1.45 mm inner diameter and in micro-channels with semi-triangular (triangular with one corner smoothed) cross-sections with hydraulic diameters of 1.09 and 1.49 mm [7,8]. They used photographs for flow regime identification and channel average voidage estimation. The typical flow regimes of bubble flow, slug flow, annular flow and churn flow were observed and their voidages were estimated [9]. Zhao and Bi investigated the co-current characteristics upward air-water two-phase flow regimes in vertical equilateral triangular channels with hydraulic diameters of 2.88, 1.44 and 0.87 mm [10]. Flow regimes were identified by both visual observations using a high-speed motion analyzer and dynamic pressure-drop measurements. Chen *et al.* investigated the characteristics of nitrogen–water two-phase flow in glass micro-channels with 1.0 mm and 1.5 mm inner diameter [11]. The two-phase flow regime, bubble speed and voidage in the micro-channels were observed and analyzed through visualization. Waelchli and Von Rohr presented flow visualization measurements of the two-phase gas–liquid flow pattern and the liquid velocity distribution inside liquid plugs of an intermittent flow. Rectangular cross-section silicon micro-channels with hydraulic diameters between 187.5 and 218 μm were tested [12]. Laser Induced Fluorescence (LIF) was used to determine the flow regime. Revellin *et al.* used optical technique to measure the frequency of bubbles generated in a microevaporator, the coalescence rates of these bubbles, their length distribution and their mean velocity, and to identify the flow regimes and their transitions in a 0.5 mm glass channel [13]. Keska and Simon applied the concomitant concentration measurements, which included a capacitive sensor, a conductive sensor, an optical system and pressure measurement devices, in a two-phase mixture in micro-channels with cross-section of 996 μm × 996 μm and 105 mm length [14]. Guet and Fortunati used a four-point optical fiber probe to measure the velocity and size of individual bubbles in a high-void fraction bubbly flow. The initial spherical equivalent diameter ranged from 4 to 10 mm, and the void fraction was up to 0.3 [15]. Although, many techniques have been used widely, the measurement of gas-liquid two phase flow in micro-pipes still needs further experimental and numerical investigation.

The electrical capacitance method is a classic and effective measurement technique that has the advantages of low cost, simple structure and non-intrusiveness, so the capacitance method has been widely used in normal scale two-phase flow systems [16–25]. However, in small-scale two-phase flow, the design, installation of capacitance sensors and the capacitance values have posed significant challenges in the application of capacitance measurement methods. Caniére *et al.* have summarized the electrode types and the applications of the capacitance methods in multiphase flow systems [26]. They found that for multiphase flow in a micro-pipe, there was no local measurement using capacitance measurements, so they designed a capacitance sensor for flow regime identification of horizontal two-phase flow in small diameter tubes with 9.0 mm inner diameter. However, use of the capacitance method for micro-pipes smaller than 9.0 mm inner diameter is seldom reported, and the performance of capacitance measurements in micro-pipe two-phase flow systems is largely unknown.

The purpose of this research is to investigate an effective and feasible way to apply the capacitance method in a micro-pipe gas-liquid two-phase flow system. As a typical flow regime, the slug flow is considered and three pipe diameters (3.96, 2.65 and 1.56 mm) are chosen in this research. The application performance and results of the capacitance measurement are discussed in detail. The voidage information is inferred from the capacitance value and the velocity of gas slug is calculated by cross-correlating two signals derived from two identical capacitance sensors.

## 2. Capacitance Measurement System

The capacitance measurement system consists of three parts: capacitance sensor, data acquisition unit and the computer. In two-phase flow, the change of the voidage and its distribution due to different permittivity of the two phases can result in variations of capacitance between the two electrodes around the pipe. The capacitance sensors are used to sense the variations in capacitance. The value of the measured capacitance reflects the voidage of the two-phase flow. The data acquisition unit is used to convert the capacitance variations to voltage signals and transmit the signals to the computer for data analysis, storage and presentation. In a capacitance measurement system, the capacitance sensor design and small capacitance measurement technique are two important aspects, which are described separately as follows.

### 2.1. Capacitance Sensor Design

**Figure 1.**
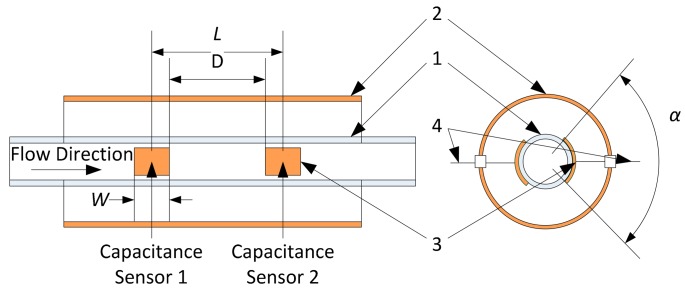
Structure of the capacitance sensor (**1**. Glass micro-pipe; **2**. Copper screen; **3**. Electrodes; **4**. Leading wires).

The structure of the capacitance sensor is an important factor in this research. Through simulation and experimental tests, the capacitance sensor as shown in Figure 1 is designed and constructed. Two identical capacitance sensors are used for gas slug velocity measurement.

Each capacitance sensor comprises a pair of electrodes (3) oppositely fixed on the outer wall of the pipe, a copper screen (2) for shielding the external noise and the shield leading wires (4). One of the electrodes is connected to an excitation voltage and the other is for capacitance detection. There are three structure parameters to be determined: the electrode length along the pipe (*W*), the electrode field angle (α) and the centre to centre distance between the two electrodes (*L*) or the nearest distance between the two electrodes (*D*).

For the electrode length W, if the local measurement of voidage is considered, the electrode length should not be too long; however, the short length makes the capacitance too small to measure. So compromising the two aspects, W is set at the values of 1.5 mm, 2.5 mm and 4.0 mm for three pipe sizes respectively, that approximately equal to pipes' inner diameter.

For the electrode field angle α, the larger the angle is, the greater the capacitance value. However, considering the production and installation of capacitance sensor, the electrode field angle is set at the range of 100°–125° in this research.

**Figure 2.**
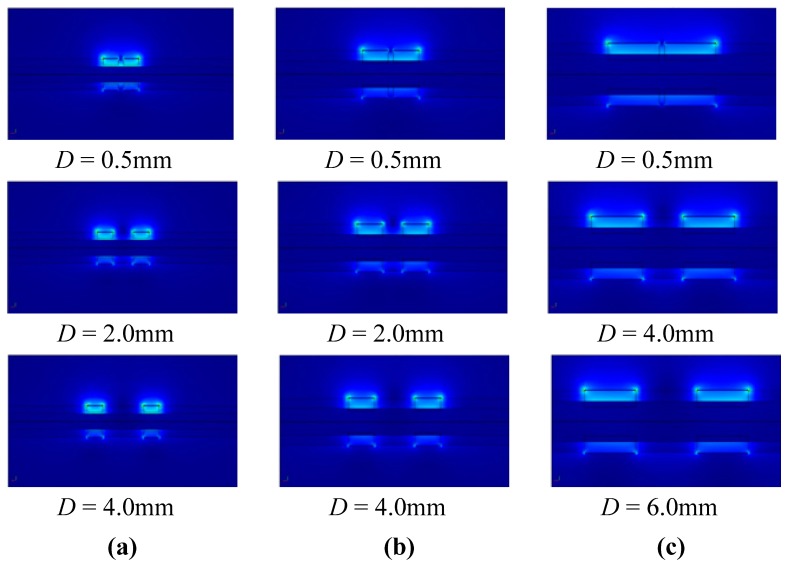
Electrical field simulations of two capacitance sensors: (**a**) Electrical field distribution in 1.56 mm inner diameter pipe with 3.0 mm outer diameter; (**b**) Electrical field distribution in 2.65 mm inner diameter pipe with 4.56 mm outer diameter; (**c**) Electrical field distribution in 3.96 mm inner diameter pipe with 6.0 mm outer diameter.

To measure the velocity of a gas slug by the cross-correlation technique, the capacitances of the two sensors must be measured simultaneously. The independence of the two capacitances must be ensured. If the two capacitance sensors were close to each other, interference between them would occur. If the spacing between them were too great, the velocity measurement error would be significant. To determine a suitable spacing between the two sensors, some computer simulation of the electrical field distribution of the capacitance sensors was carried out. Finite element method (FEM) is used for the simulation [26,27]. The electrical potential, field distribution and capacitance can be calculated using the following equations:

(1) $$-\nabla \cdot (\varepsilon (\vec{x})\nabla u(\vec{x}))=\rho (\vec{x})$$

(2) $$\vec{E}\vec{\left(x\right)}=-\nabla u(\vec{x})$$

(3) $$C=\frac{1}{U}\int_{S}\varepsilon_{0}\varepsilon_{r}(\vec{E}\cdot \vec{n})dS$$

where *x⃗* is the space vector, ε(*x⃗*) is the dielectric constant, *u*(*x⃗*) is the potential function, ρ(*x⃗*) is the charge density function, *E⃗*(*x⃗*) is the electrical field vector, U is the voltage between the source and detector electrode, ε_0_ = 8.8542 × 10^−12^ *F*/*m* is the dielectric constant in the free space, ε*_r_* is the relative dielectric constant, *S* is a closed curve enclosing the detector electrode, and *n⃗* is the unit normal vector to *S*. Typical results obtained are shown in Figure 2.

Figure 2 shows the electrical field distribution for different distances *D* between the two capacitance sensors on three different pipe sizes. According to the simulation results, when the distance *D* is larger than the outer diameter of the pipe, the electrical field distribution is uniform, and the interference between two capacitances is very small. The spacing between the sensors *D* is chosen to be 8.0, 4.5 and 3.5 mm for 3.96, 2.65 and 1.56 mm inner diameter pipes, respectively. The structure parameters of the capacitance sensor are summarized in Table 1.

### 2.2. Measurement Circuit

**Figure 3.**
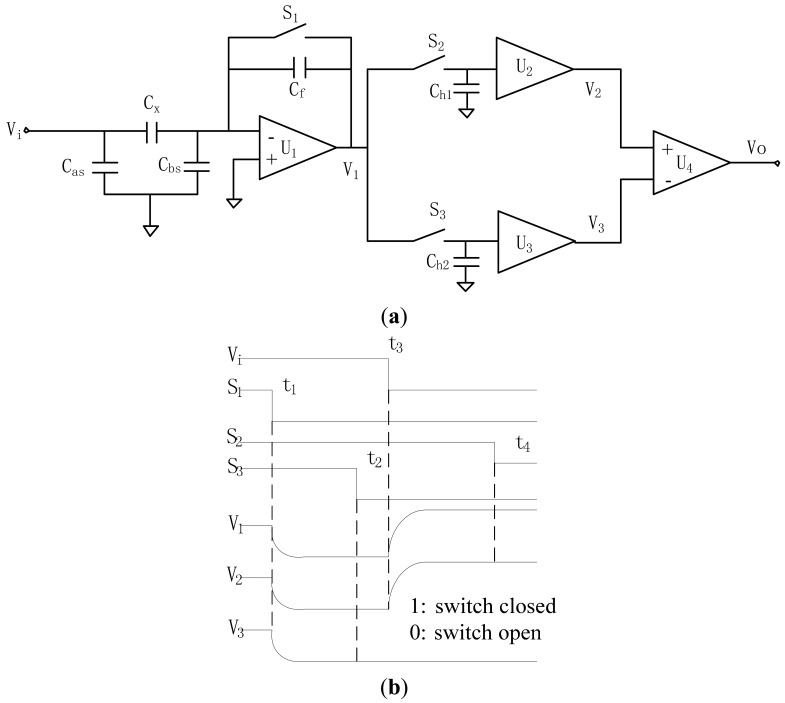
(**a**) Principle of the capacitance measuring circuit; (**b**) Time sequence of measurement circuit.

The techniques suitable for industrial measurement of capacitance include resonance, oscillation, charge/discharge and AC bridge methods [28–32]. In this paper, the capacitance measurement circuit based on a high-speed differential sampling method is used, which operates in the way of charging/discharging and yields a voltage which is proportional to the measured capacitance [33]. The principle of the circuit and the time sequence of the control and output signals of amplifiers is shown in Figure 3.

In Figure 3a, *V_i_* is the voltage of the exciting source. The charge amplifier consists of an amplifier *U*_1_, capacitor *C _f_* and switch *S*_1_. Switches *S*_2_ and *S*_3_ with voltage followers *U*_2_ and *U*_3_ and hold capacitors *C_h_*_1_ and *C_h_*_2_ form two sample/holders (*S*/*Hs*). The circuit operation process can be divided into two main steps. The first step is to measure the charge injection effect of switch *S*_1_. At the start of a typical operating cycle, *V_i_* is high and *S*_1_ is closed. Both S/Hs are in a sample mode. The output of *U*_1_ is 0 V because *S*_1_ is closed. At the time of *t*_1_,*S*_1_ is opened, and *V*_1_, ideally, would keep 0 V, but due to the charge-injection effect, an amount of charge *Q_c_* is injected into *C_f_*. It will make *V*_1_ down to *V*_L_. At the time of *t*_2_, the output of *U*_1_ is stable and the output of *U*_3_,*V*_3_, equals *V*_L_, and *S*_3_ is opened to keep the *S*/*H* in hold mode. In the second step the charge injected into *C_x_* is measured. At the time of *t*_3_, a high-to-low transition occurred at the excitation source *V_i_*, and the charge on the left electrode of *C_x_* is:

(4) $$Q=\Delta V_{i}C_{x}$$

and the output of *U*_1_ is:

(5) $$V_{H}=V_{L}-\frac{Q}{C_{f}}$$

At the time of *t*_4_,*S*_2_ is opened to hold the voltage *V_H_* at the output of *U*_2_,*V*_2_, and the output of the instrument amplifier *U*_4_ is:

(6) $$V_{O}=V_{H}-V_{L}=\frac{-\Delta V_{i}C_{x}}{C_{f}}$$

Apparently, voltage *V_o_* is proportional to the measured capacitance (*C_x_*).

It should be stressed that this capacitance measuring circuit is inherently stray immune, *i.e.*, the circuit is insensitive to the stray capacitances (*C_as_* and *C_bs_*, the capacitances between the electrodes and ground). As shown in Figure 3b, *C_as_* is driven by the excitation source and has no influence on the current through the measured *C_x_*. The stray capacitance *C_bs_* is kept at virtual ground by the op-amps and there is no potential difference across *C_bs_*. Hence, *C_bs_* also has no effect on the capacitance measurement. The capacitance measurement circuit has a resolution of 0.5 fF with a sample frequency up to 100 kHz, which can satisfy the requirements for high-speed measurement of small capacitance.

## 3. Experimental Setup

**Figure 4.**
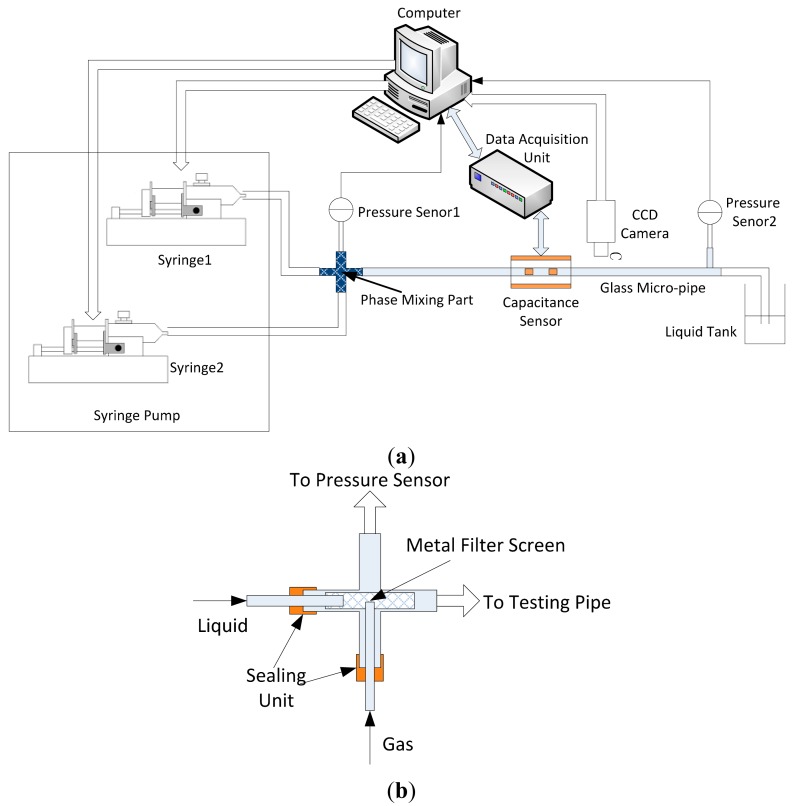
(**a**) Layout of the experimental setup; (**b**) Structure of the mixing part.

The layout of the experimental setup is shown in Figure 4. The setup is composed of a syringe pump, mixing part, testing pipe, pressure sensors and capacitance measurement system. A Model 33 syringe pump (Harvard Apparatus Inc., Boston, MA, USA) is used to drive gas and liquid. The syringe pump can implement dual syringe infusion or withdrawal and parallel or reciprocal motion. The range of flowrate is from 0.0073 μL/h (using a 10 μL syringe) to 53.346 mL/min (with a 50 mL syringe). The accuracy can reach ±0.35%. The horizontal glass pipes on which tests were carried out are shown in Table 1. The structure of the mixing part is very important. Different designs can result in different mixing effects, and produce different flow regimes. The structure scheme of the mixing part shown in Figure 4b is applied. The metal filter screen is used in this design. The gas and liquid phases are injected into the metal filter screen directly. The full mixture using the filter screen can produce bubble flow, slug flow and annular flow. The experimental results show that the applied mixing part is effective. Two MPX 10 DP micro differential pressure sensors (Motorola, Chicago, IL, USA) are adopted for pressure monitoring. The CCD camera is used to record the motion image continuously for monitoring flow regime and validating the experimental results.

The fluids used in the experiments are air as gas phase and glycerol as liquid phase. The relative dielectric constants of air and glycerol are 1 and 45.8, respectively. The conductivity of glycerol is about 6.4 × 10^−8^ s/cm (25 °C). The sample frequency of the capacitance measurement system is set at 2.6 kHz. In experiments, the range of liquid flowrate is from 0.1 to 20.0 mL/min, and the range of gas flowrate is from 1.0 to 30.0 mL/min.

## 4. Results and Discussion

### 4.1. Capacitance Signal Analysis and Voidage Measurement

During experiments, the empty pipe capacitance value *C*_0_ (when the pipe is filled with air) and the full pipe capacitance values *C_m_* (when the pipe is filled with liquid) are measured. Because the installation of the capacitance sensor and the operation condition are not totally same, *C*_0_ and *C_m_* are different for the two capacitance sensors. So the measurement of *C*_0_ and *C_m_* is necessary for calibrating the sensors.

**Figure 5.**
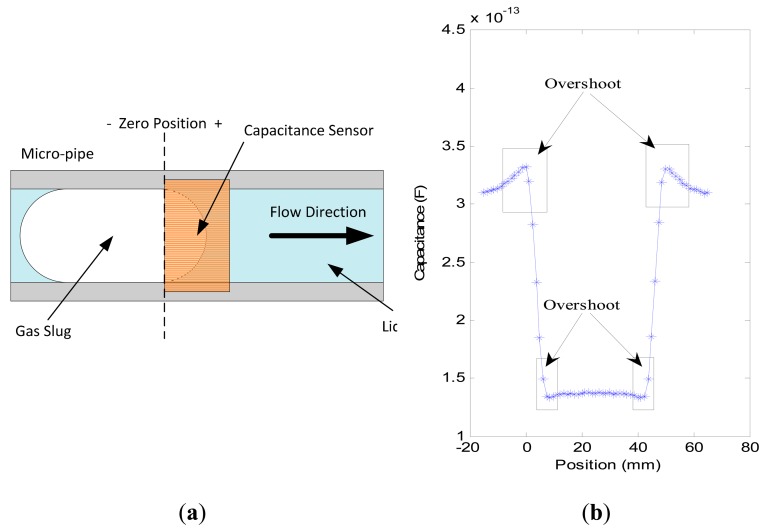
(**a**)The ideal gas slug passing through the capacitance sensor; (**b**) Simulation results of a gas slug passing through the capacitance sensor.

Figure 5 shows the simulation of ideal gas slug passing through capacitance sensor. Figure 5a is the sketch figure of an ideal gas slug passing through capacitance sensor. The length of gas slug is set to 45.0 mm, and the pipe with an inner diameter of 3.96 mm is selected to implement the simulation. The length of electrode is 5.0 mm. *C*_0_ is about 137.6 fF, and *C_m_* is about 306.1 fF. In Figure 5b, the x-coordinate is the position of gas slug head, and zero is the starting point of the capacitance sensor, as shown in Figure 5a.

**Figure 6.**
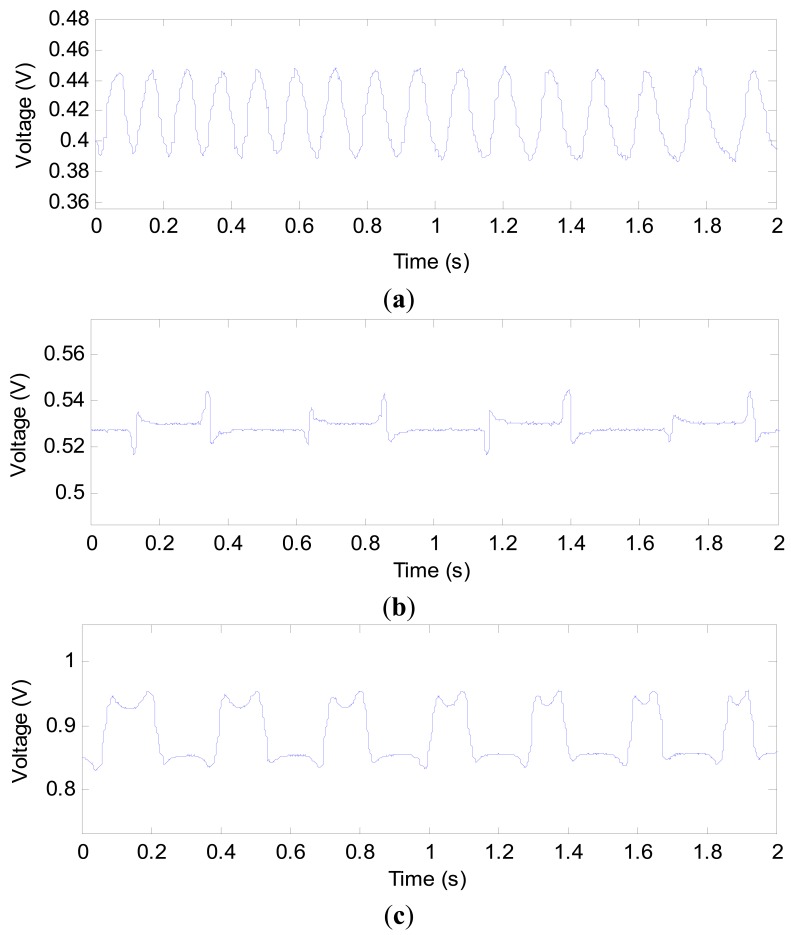
(**a**) Signal from capacitance sensor in 1.56 mm inner diameter pipe with 10.0 mL/min gas flowrate and 1.0 mL/min liquid flowrate; (**b**) Signal from capacitance sensor in 2.65 mm inner diameter pipe with 10.0 mL/min gas flowrate and 10.0 mL/min liquid flowrate; (**c**) Signal from capacitance sensor in 3.96 mm inner diameter pipe with 30.0 mL/min gas flowrate and 10.0 mL/min liquid flowrate.

In Figure 5b, the variations in capacitance can be clearly observed when the gas slug passes through the capacitance sensor. However, an overshoot in the measured capacitance is evident, that is, the capacitance value is beyond *C_m_* or below *C*_0_. For example, in Figure 5b, the maximum value is 332.0 fF, and the minimum value is 132.9 fF. This phenomenon occurs at the measurement area where the phases begin to change, for example, from gas phase to liquid phase or *vice versa*. The practical experiments also verify the phenomenon, as shown in Figure 6. The overshoot is due to the characteristics of the sensing field of the capacitance sensor, which is non-uniformly distributed in the cross-section and in the axial direction. In some regions, the sensitivity exhibits a positive response, otherwise it is negative [34,35]. Figure 6 shows some practical experimental results. As an instance, the capacitance signals from sensor 2 are presented for every pipe.

**Figure 7.**
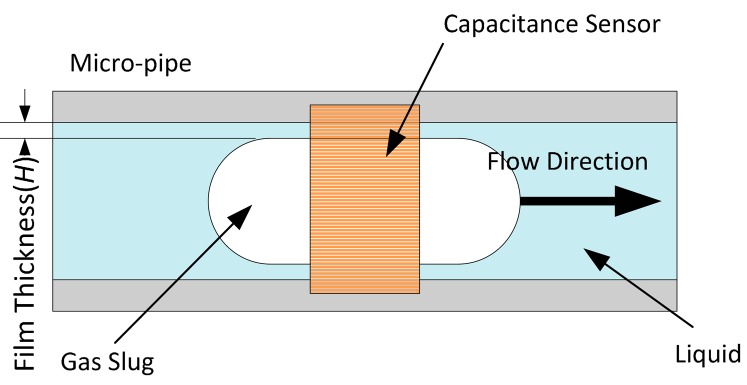
The film thickness between gas phase and the inner wall of micropipe.

The three waveforms in Figure 6 are very similar to the one in Figure 5, and the overshoot still exists. Though the overshoot will affect the voidage measurement, the signal contains the information about the voidage and reflects its variation. The accurate estimation of voidage need be further investigated. The experimental results also show that when a gas slug is located at the position of the capacitance sensor, the capacitance value is greater than that of the empty pipe, *i.e.*, a liquid film exists between the gas phase and inner wall of the pipe, as shown in Figure 7.

Previous research has demonstrated this phenomenon, and in some cases, the thickness of the liquid film will increase with the capillary number *C_a_* = μν/σ where μ and *ν* are the viscosity and velocity of the liquid phase respectively and σ is liquid surface tension [36–38]. In the experiments, the measured capacitance signal can reflect the variation of the liquid film thickness of the gas slug. The faster the gas slug flows, the thicker the liquid film is.

### 4.2. Gas Slug Velocity Measurement

Velocity is another important parameter in a two-phase flow system. In this research, the cross-correlation technique is used to implement the velocity measurement. Two identical capacitance sensors (upstream sensor and downstream sensor) are mounted along the micro-pipe, as shown in Figure 4. The capacitance signals of the upstream and downstream sensors are used to calculate the transit time, which can reflect the average time delay between the two signals. Initially, the original capacitance signal is normalized by the following equations:

(7) $$C_{x1}^{'}=\frac{C_{x1}-C_{01}}{C_{m1}-C_{01}}$$

(8) $$C_{x2}^{'}=\frac{C_{x2}-C_{02}}{C_{m2}-C_{02}}$$

(9) $$E_{x1}=C_{x1}^{'}-\bar{C_{x1}^{'}}$$

(10) $$E_{x2}=C_{x2}^{'}-\bar{C_{x2}^{'}}$$

where *C_x_*_1_ and *C_x_*_2_ are the capacitance values obtained from the two sensors respectively, *C*_01_ and *C*_02_ are the capacitance values of the empty pipe, respectively, *C_m_*_1_ and *C_m_*_2_ are the capacitance values when the pipe is full of liquid, respectively, $C_{x1}^{\prime }$ and $C_{x2}^{\prime }$ are the normalized values, *E_cx_*_1_ and *E_cx_*_2_ are the normalized values with zero mean. Then the cross-correlation algorithm is used to calculate the transit time τ:

(11) $$\begin{array}{cc} R_{E_{x1}E_{x2}}(j)=\frac{1}{N}\sum_{n=1}^{N}E_{x1}(n)E_{x2}(n+j), & j=0,1,2,\ldots ,J \end{array}$$

According to Equation (11), the max value of *R_Ex_*_1_*_Ex_*_2_(*K*) can be found, then τ = *K* × Δ*t* can be obtained, where Δ*t* is the internal time of sampling. Finally, the gas slug velocity can be calculated from the following equation:

(12) $$\nu =\frac{L}{\tau }$$

**Figure 8.**
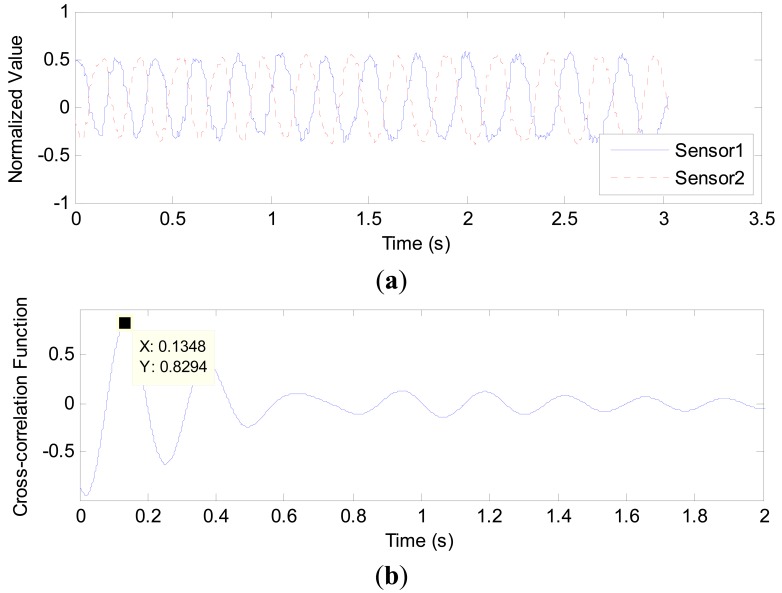
Signal waveforms and the resulting correlation function in 1.56 mm inner diameter pipe with 10.0 mL/min gas flowrate and 1.0 mL/min liquid flowrate: (**a**) Normalized signals from the two capacitance sensors; (**b**) Cross-correlation function between the two signals in (a).

**Figure 9.**
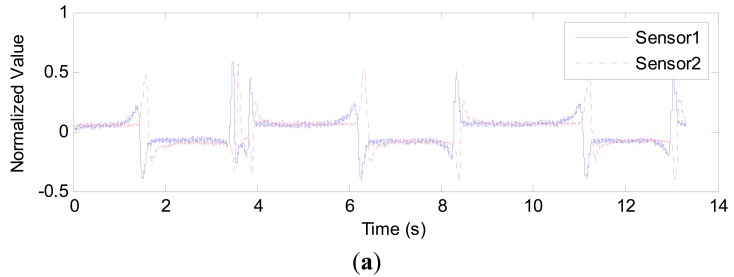

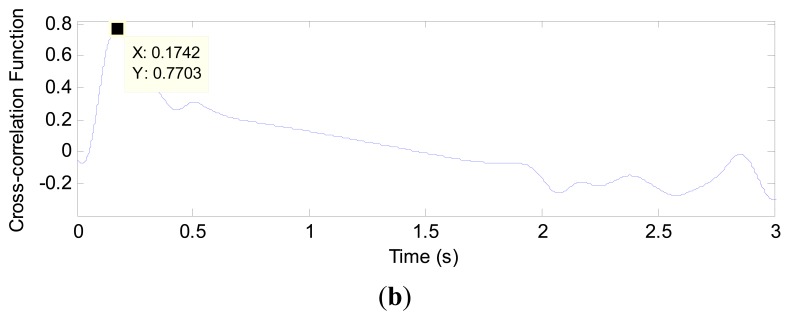
Signal waveforms and the resulting correlation function in 2.65 mm inner diameter pipe with 4.0 mL/min gas flowrate and 4.0 mL/min liquid flowrate: (**a**) Normalized signals from the two capacitance sensors; (**b**) Cross-correlation function between the two signals in (a).

**Figure 10.**
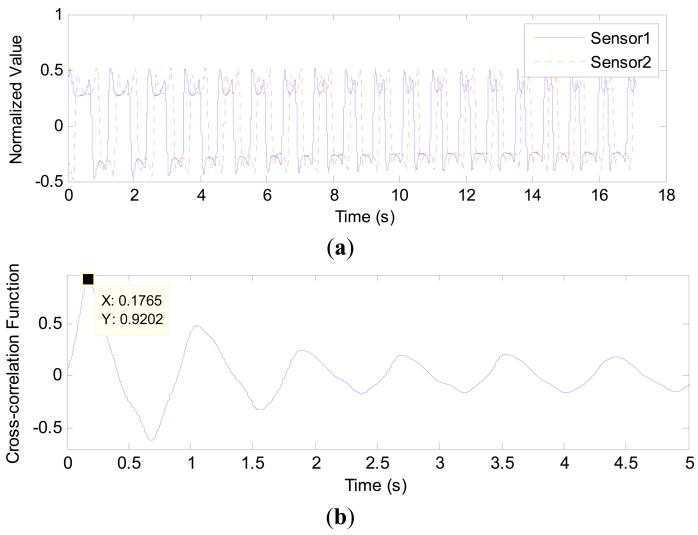
Signal waveforms and the resulting correlation function in 2.65 mm inner diameter pipe with 4.0 mL/min gas flowrate and 4.0 mL/min liquid flowrate: (**a**) Normalized signals from the two capacitance sensors; (**b**) Cross-correlation function between the two signals in (a).

Figures 8, 9 and 10 show some results obtained from the 1.56, 2.65 and 3.96 mm inner diameter pipes, respectively. In each figure, sub-figure (a) is the curve of two normalized capacitance signals; sub-figure (b) is the cross-correlation function between the two signals, from which the transit time is determined. In Figure 8, the transit time τ is 0.1348 s and the centre to centre distance between the two sensors *L* is 5.0 mm, so the gas slug velocity is 0.0371 m/s. Similarly, in Figure 9, the velocity of gas slug is 0.0402 m/s whilst in Figure 10, the velocity is 0.0737 m/s.

**Figure 11.**
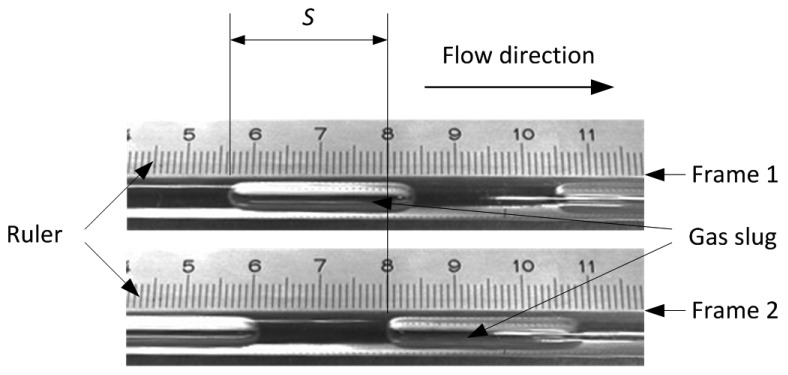
The images of gas slugs passing through the testing pipe.

Figure 11 shows two images of a gas slug flowing through the testing pipe at different times, which is recorded by a CCD camera for velocity calibration. The motion distance of the gas slug (*S*) can be easily obtained using a ruler as shown in Figure 11. The interval time between the two images (*t*) can be obtained from the sampling frequency of the CCD camera. Then the velocity of the gas slug can be calculated. The measured velocity through the cross-correlation technique is 0.0371 m/s (*L* = 13.0 mm, τ = 0.35 s), and the calibration velocity is 0.0383 m/s (*S* = 23.0 mm, *t* = 0.6 s).

**Figure 12.**
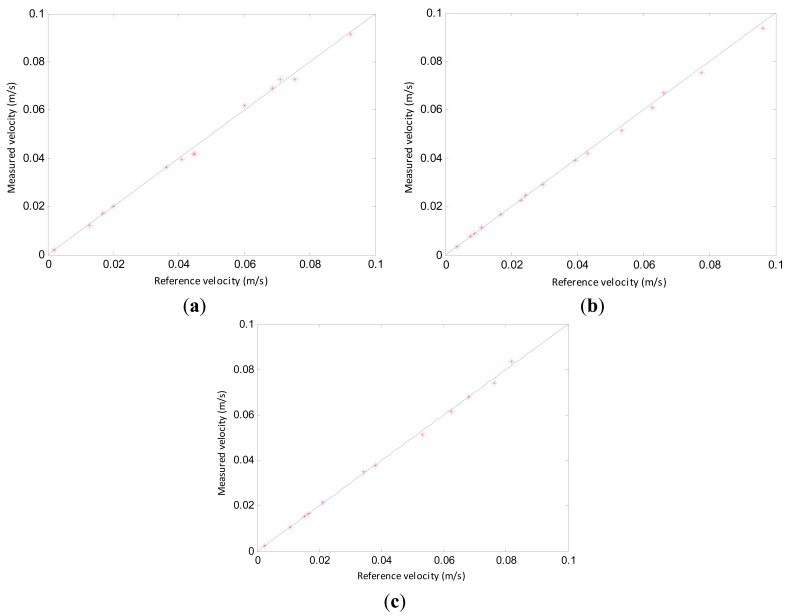
Comparison between the measured and reference velocities: (**a**) Experimental results with 1.56 mm inner diameter pipe; (**b**) Experimental results with 2.65 mm inner diameter pipe; (**c**) Experimental results with 3.96 mm inner diameter pipe.

Figure 12 shows a comparison between the measured velocity using the cross-correlation technique and the reference velocity. The measured velocity is consistent with the reference velocity. The results indicate that the cross-correlation velocity measurement method is effective and can be used to measure the velocity of gas slugs.

## 5. Conclusions

In this research, a simple capacitance sensor is developed for the measurements of void fraction and slug velocity in a gas-liquid flow. The parameters of the capacitance sensor are optimized and verified through computer simulation. Based on the high-speed differential sampling method, a high-resolution and high-speed capacitance measurement circuit is adopted. The circuit has a resolution of 0.5 fF with a sample frequency up to 100 kHz. The following conclusions can be drawn from the results presented:

(1) The measured capacitance signal can reflect the voidage variations and can be used to measure cross-sectional voidage of gas-liquid two-phase flow.
(2) The overshoot phenomenon in capacitance signal measurement occurs not only in practical experiments, but also in simulation results. Further research will be carried out to study this phenomenon.
(3) The capacitance signal can reflect the variation of the liquid film thickness of gas slug, which shows that the liquid film between the gas phase and the inner wall of the glass pipe increases with the capillary number that is proportional to slug flow velocity.
(4) By comparing with CCD camera calibration results, the cross-correlation method is verified to be effective. Using it, the velocity of gas slugs can be calculated correctly.

The simulation and experimental results have demonstrated that the capacitance sensor is effective and feasible for the parameter measurement of gas-liquid two-phase flow in micro-pipes.

## Figures and Tables

**Table 1.** Structure parameters of the capacitance sensor.

**Inner Diameter (mm)**	**Outer Diameter (mm)**	**Length of Pipe (mm)**	***W*(mm)**	**α**	***D*(mm)**	***L*(mm)**
1.56	3.0	300	1.5	106°	3.5	5.0
2.65	4.5	500	2.5	122°	4.5	7.0
3.96	6.0	500	5.0	125°	8.0	13.0
